# Toll-like receptor 2 induced cytotoxic T-lymphocyte-associated protein 4 regulates *Aspergillus*-induced regulatory T-cells with pro-inflammatory characteristics

**DOI:** 10.1038/s41598-017-11738-4

**Published:** 2017-09-13

**Authors:** Ruud P. H. Raijmakers, Evelien G. G. Sprenkeler, Floor E. Aleva, Cor W. M. Jacobs, Thirumala-Devi Kanneganti, Leo A. B. Joosten, Frank L. van de Veerdonk, Mark S. Gresnigt

**Affiliations:** 1 Department of Experimental Internal Medicine and Radboud Center for Infectious diseases (RCI), Radboud University Medical Center, Geert Grooteplein zuid 8, 6525GA Nijmegen, The Netherlands; 2 Department of Respiratory Medicine, Radboud University Medical Center, Geert Grooteplein zuid 10, 6525 GA Nijmegen, The Netherlands; 3 Department of Immunology, St. Jude Children’s Research Hospital, 262 Danny Thomas Place, Memphis, TN 38105 USA

## Abstract

Patients with cystic fibrosis, chronic obstructive pulmonary disease, severe asthma, pre-existing pulmonary lesions, and severely immunocompromised patients are susceptible to develop infections with the opportunistic pathogenic fungus *Aspergillus fumigatus*, called aspergillosis. Infections in these patients are associated with persistent pro-inflammatory T-helper (T_H_)2 and T_H_17 responses. Regulatory T-cells, natural suppressor cells of the immune system, control pro-inflammatory T-cell responses, but can also contribute to disease by shifting to a pro-inflammatory T_H_17-like phenotype. Such a shift could play an important role in the detrimental immunopathology that is seen in aspergillosis. Our study demonstrates that *Aspergillus fumigatus* induces regulatory T-cells with a T_H_17-like phenotype. We also demonstrate that these regulatory T-cells with a pro-inflammatory T_H_17-like phenotype can be reprogrammed to their “classical” anti-inflammatory phenotype by activating Toll-like receptor 2 (TLR2), which regulates the induction of cytotoxic T-lymphocyte-associated protein 4 (CTLA4). Similarly, soluble CTLA4 could reverse the pro-inflammatory phenotype of *Aspergillus-*induced regulatory T-cells. In conclusion, our results suggest a role for regulatory T-cells with a pro-inflammatory T_H_17-like phenotype in *Aspergillus*-associated immunopathology, and identifies key players, i.e. TLR2 and CTLA4, involved in this mechanism.

## Introduction

Opportunistic infections caused by *Aspergillus fumigatus* are frequently observed in immunocompromised patients. These infections are considered to be severe complications and result from the absence of a fully functional host defence, thereby increasing susceptibility to this fungus^1^. Other patient groups at risk of developing disease caused by *A. fumigatus* are patients with cystic fibrosis (CF), chronic obstructive pulmonary disease (COPD), severe asthma, or individuals with pre-existing pulmonary lesions^2–6^. Clinical manifestations of such disease are called (invasive) aspergillosis, and range from hypersensitivity reactions to *A. fumigatus*, as is seen in allergic bronchopulmonary aspergillosis (ABPA)^5, 7^, to insufficient clearance of *A. fumigatus* with long-lasting inflammatory responses and ongoing fungal growth, as is seen in chronic pulmonary aspergillosis (CPA)^2, 3^.

Adequate clearance of *A. fumigatus* relies on T-helper cell-mediated pro-inflammatory immune responses, and particularly the T-helper (T_H_)1 response^8–11^. However, T-helper responses, in particular T_H_2 and T_H_17, are also known to play a detrimental role in the pathogenesis of ABPA and CPA^12–14^. These responses can cause uncontrolled inflammation, resulting in a massive influx of eosinophils and neutrophils^12, 15^. Although T_H_17-mediated recruitment of neutrophils plays an important role in the clearance of fungi, this response can also play a detrimental role in protective immunity during aspergillosis^10, 11^. T_H_17 activation by fungal growth can lead to disruption and necrosis of pulmonary tissue, thereby creating a niche for saprophytic growth of *A. fumigatus* ^1^, allowing the fungus to persist within the lungs and continue to induce persistent inflammatory responses^16^.

An important inhibitor of T-helper cell-driven pro-inflammatory responses are the regulatory T (T_reg_) cells. This endogenous immune suppressive cell controls T_H_2 polarization, and neutralizes T_H_17 responses in the lungs^17^. Adoptive transfer of T_reg_ cells, or localised over-expression of Forkhead box protein P3 (FoxP3) (the classical transcription factor of T_reg_ cells), inhibits T_H_2 and T_H_17 responses in animal models for asthma, resulting in improvement of clinical scores^18, 19^. T_reg_ cells are known to modulate immune responses through both contact dependant (cytotoxic T-lymphocyte-associated protein 4; CTLA4, Indoleamine-pyrrole 2,3-dioxygenase; IDO, membrane bound Transforming growth factor β; TGFβ, and competition for major histocompatibility complex; MHC), and contact independent (Interleukin-10 and TGFβ) signalling pathways^20, 21^.

Recently, several studies demonstrated that T_reg_ cells can acquire pro-inflammatory characteristics by differentiating into Interleukin (IL)-17A producing RAR-related orphan receptor γt (RORγt) expressing T-cells^22, 23^. This conversion was found to be involved in the pathogenesis of autoimmune arthritis and depends on pro-inflammatory cytokines such as IL-2 and IL-15^23^. T_H_17 differentiation promoting cytokines, like IL-1β, IL-6, and IL-23 further influence this conversion^22^. Interestingly, in CPA patients, IL-1 and IL-15 cytokine levels were found to be upregulated, suggesting a T_reg_ – T_H_17 interplay in these patients^14^. Such a shift could play a crucial role in the IL-17 mediated immunopathology that is seen in patients with aspergillosis.

If *A. fumigatus* is capable of inducing T_reg_ cells with a pro-inflammatory phenotype, this could have important implications for our understanding of the detrimental immunopathology seen in aspergillosis. In that case, reversal of pro-inflammatory T_reg_ cells to their “classical” anti-inflammatory state could be a promising strategy for immunomodulatory therapy. This study shows that human *A. fumigatus*-induced T_reg_ cells can exert this pro-inflammatory phenotype. In addition, we investigate the mechanisms involved and the potential of T_reg_ cells to be reprogrammed to their “classical” anti-inflammatory phenotype.

## Results

### *A. fumigatus* induces regulatory T-cells with a pro-inflammatory T_H_17-like phenotype

**Figure 1:**
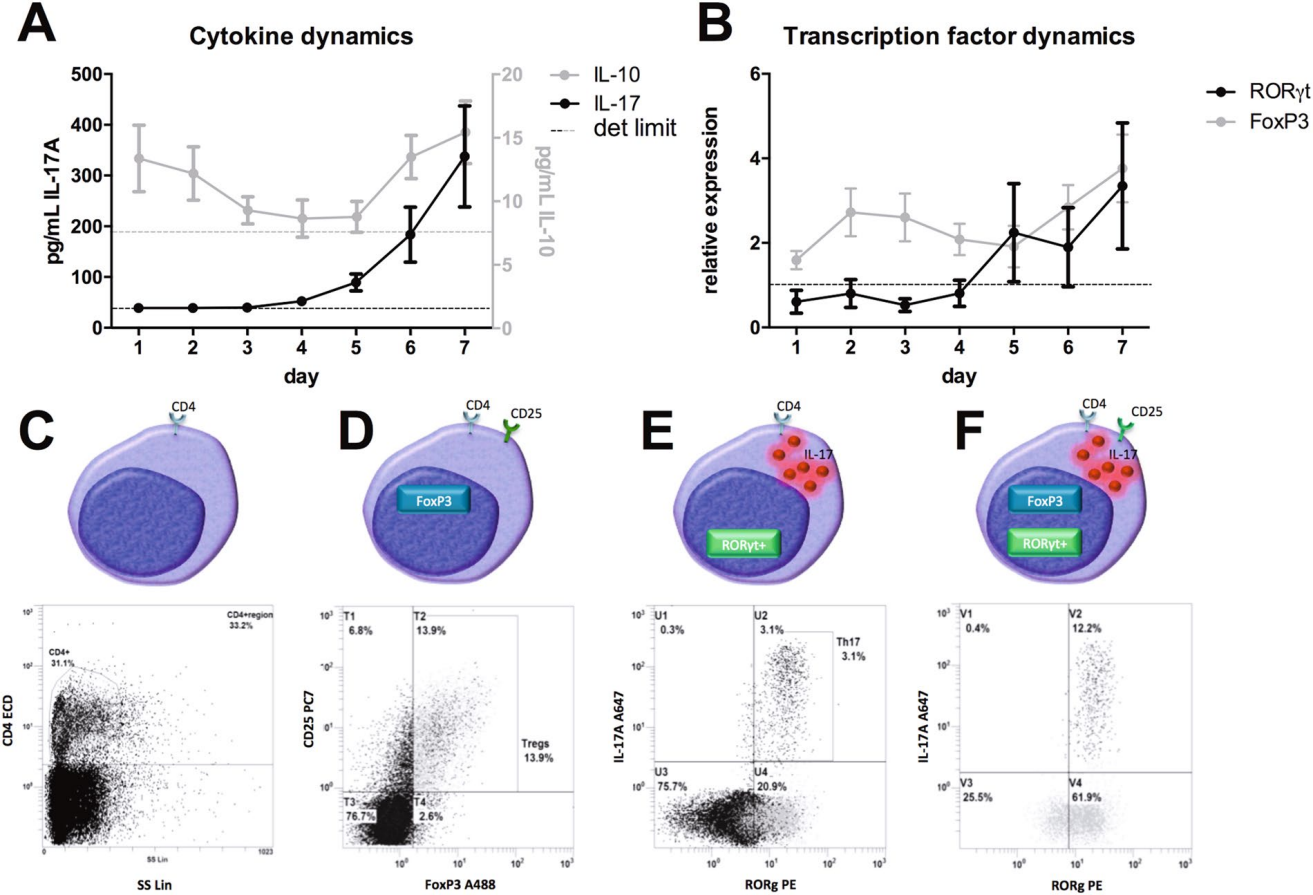
Expression markers for regulatory T-cells and T_H_17 cells. (**A**) Dynamics of IL-17 and IL-10 cytokine levels in culture supernatants of PBMCs exposed to heat-inactivated *A. fumigatus* conidia (1 × 10^7^/mL) for 7 days. (**B**) Dynamics of RORγt and FoxP3 mRNA expression in PBMCs exposed to *A. fumigatus* conidia for 7 days. (**C**–**F**) Gating strategy to detect regulatory T-cells with T_H_17 characteristics. (**C**) T-helper cells were selected by expression of the marker CD4 (CD4^+^), (**D**) regulatory T-cells were selected within the CD4+ population using the characteristics (CD25^+^ FoxP3^+^), (**E**) T_H_17 cell were selected within the CD4+ population using the characteristics (RORγt^+^ IL-17A^+^), and (**F**) T_H_17 cell characteristics (RORγt^+^ IL-17A) were selected within regulatory T-cells (CD4^+^ CD25^+^ FoxP3^+^) upon stimulation with heat-inactivated *A. fumigatus* conidia (1 × 10^7^/mL).

By determining the kinetics of IL-17A and IL-10 production over a course of 7 days in PBMCs simulated with *A. fumigatus* conidia, we determined the optimal time point to detect T_H_17-like pro-inflammatory T_reg_ cells. Similar to previous studies with *Candida albicans* ^24^, *A. fumigatus*-induced IL-17A release was detectable at peak levels in supernatants after 7 days (Fig. 1A). The production of IL-10, an anti-inflammatory cytokine required for T_reg_ cell differentiation^25^, which is also produced by T_reg_ cells, demonstrated a biphasic response (Fig. 1A). Expression of the transcription factor of T_H_17 cells, RORγt, started to increase after 5 days exposure to conidia, similar to IL-17A release, whereas FoxP3 expression was already induced after 24 h and continued to increase in expression towards 7 days (Fig. 1B).

To detect *A. fumigatus*-induced T_H_17-like pro-inflammatory T_reg_ cells, PBMCs were stained and measured by flowcytometry after 7 days of stimulation with *A. fumigatus* conidia. T-cells were identified through CD4 (Fig. 1C). Within the CD4^+^ population, the number of T_reg_ cells was quantified as the percentage of CD25^+^FoxP3^+^ cells (Fig. 1D). T_H_17 cells were quantified as RORγt^+^IL-17A^+^ cells within the CD4^+^ population (Fig. 1E). Finally, the percentage of cells with T_H_17 markers, i.e. RORγt / IL-17A, was determined within the T_reg_ population, i.e. CD25^+^ FoxP3^+^ (Fig. 1F).

**Figure 2:**
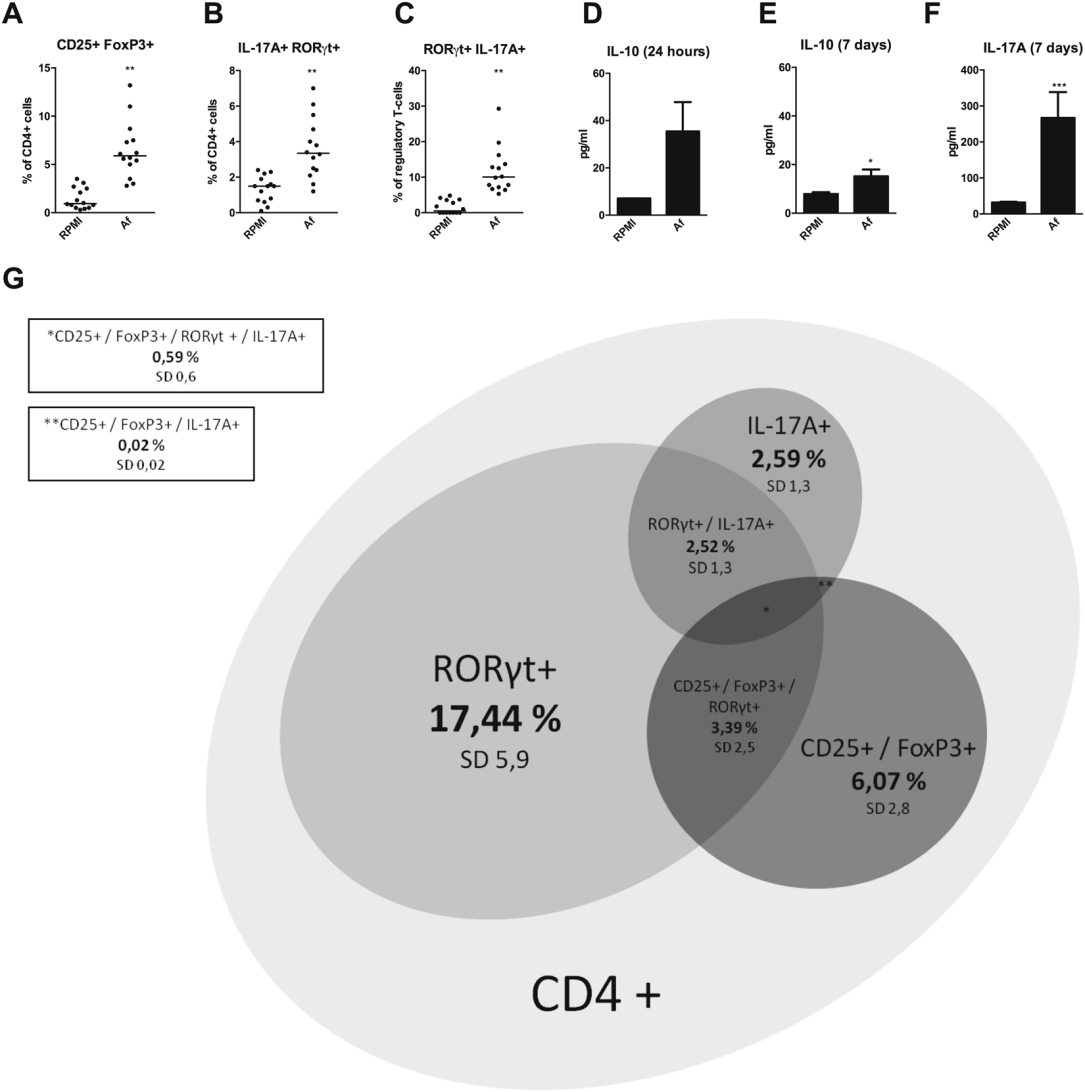
*Aspergillus* induces regulatory T-cells with a T_H_17-like phenotype. Scatter plots with median showing (**A**) Regulatory T-cell (CD25^+^ FoxP3^+^) induction after 7 days in human PBMCs stimulated with either RPMI, or heat-inactivated *A. fumigatus* conidia (1 × 10^7^/mL). (**B**) IL-17A and RORγt expression within regulatory T-cells after 7 days in human PBMCs stimulated with either RPMI, or heat-inactivated *A. fumigatus* conidia (1 × 10^7^/mL). (**C**) T_H_17 cell (IL-17A^+^ RORγt^+^) induction after 7 days in human PBMCs stimulated with either RPMI, or heat-inactivated *A. fumigatus* conidia (1 × 10^7^/mL) (n = 17 donors). (**D**) IL-10 production after 24 hours in human PBMCs (n = 5 donors) stimulated with either RPMI, or heat-inactivated *A. fumigatus* conidia (1 × 10^7^/mL). (**E**) IL-10 and (**F**) IL-17A production after 7 days in human PBMCs (n = 17 donors) stimulated with either RPMI, or heat-inactivated *A. fumigatus* conidia (1 × 10^7^/mL). (**G**) Proportional Venn diagram showing overlap in CD4 cell-phenotype after PBMCs (n = 17 donors) were stimulated with heat-inactivated *A. fumigatus* conidia (1 × 10^7^/mL) for 7 days. Results are depicted as percentage of CD4^+^ cell population with concomitant standard deviation (SD). Data are represented as scatter dot plots with median. Abbreviations: *Af* = *Aspergillus fumigatus*. *p-value ≤ 0.05, **p-value ≤ 0.01,***p-value ≤0.001.

Following stimulation with *A. fumigatus*, a significant induction of CD25^+^FoxP3^+^ T_reg_ cells (p = 0.0017 n = 14) (Fig. 2A), RORγt^+^IL-17A^+^ T_H_17 cells (p = 0.0017 n = 14) (Fig. 2B), and CD25^+^FoxP3^+^ RORγt^+^IL-17A^+^ pro-inflammatory T_reg_ cells (p = 0.0011 n = 14) (Fig. 2C) was observed. The observed overlap between regulatory T-cell phenotype (CD25^+^FoxP3^+^), and T_H_17 cell-phenotype (RORγt^+^, IL-17A^+^, or RORγt^+^IL-17A^+^), within the CD4^+^ cell-population, following stimulation with *A. fumigatus*, is depicted as a Venn diagram in Fig. 2G.

In order to assess the cytokine release by these different cell populations, IL-10 production was measured in the culture supernatant after 24 hours and 7 days, and IL-17A production was measured after 7 days. After 7 days of stimulation, production of both IL-10 and IL-17A was significantly increased (p = 0.0273 n = 15 and p < 0.0001, n = 17) (Fig. 2E and F). However, this effect was not observed for IL-10 after 24 hours (Fig. 2D).

### TLR2 regulates regulatory T-cells with a T_H_17-like phenotype

Toll-like receptor (TLR)2 is involved in the recognition of pathogen-associated molecular patterns (PAMPS) in the *Aspergillus* cell wall (reviewed in ref. 26), and is associated with the induction of T_reg_ cells in response to fungi^27, 28^. Based on the observation that naïve splenocytes of *Tlr2* ^−/−^ mice produce higher levels of IL-17A than wild type (WT) mice (Supplementary Figure 1), we hypothesized that TLR2 could have a role in shaping the population of *Aspergillus*-induced regulatory T-cells.

To investigate the role of TLR2 in the induction of T_reg_ cells with a T_H_17-like phenotype, human PBMCs were stimulated with *A. fumigatus* conidia for 24 hours and 7 days while TLR2 was blocked with a neutralizing antibody.

**Figure 3:**
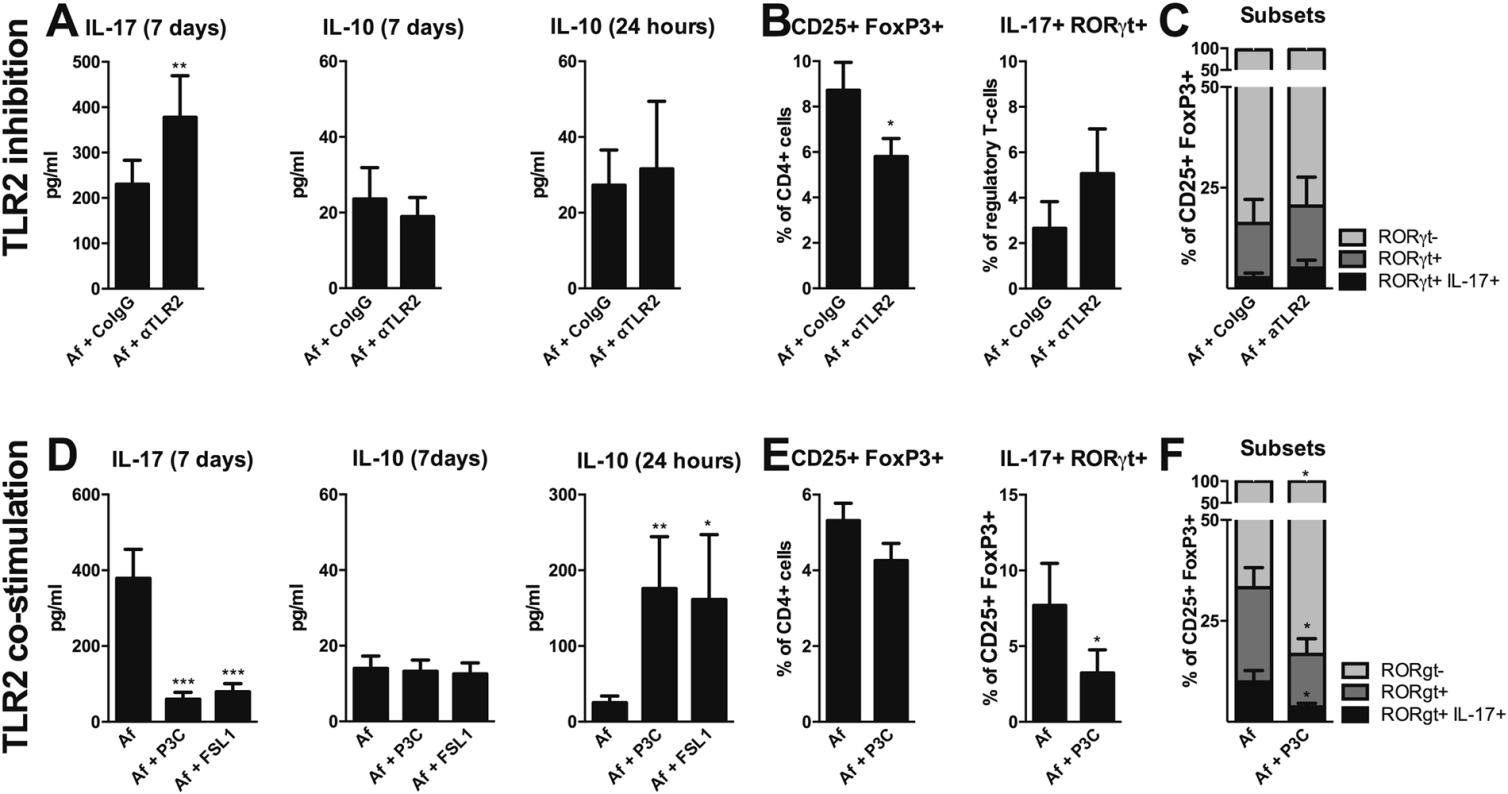
TLR2 regulates *Aspergillus*-induced regulatory T-cells with a T_H_17-like phenotype. (**A**) IL-17A production after 7 days, IL-10 production after 24 hours and 7 days, regulatory T-cell (CD25^+^ FoxP3^+^) induction after 7 days, and IL-17A and RORγt expression within regulatory T-cells after 7 days in human PBMCs (n = 6 donors) stimulated with heat-inactivated *A. fumigatus* conidia (1 × 10^7^/mL) after 1 hour pre-incubation with 10 µg/mL αTLR2, or CoIgG. (**B**,**C**) Different subsets within regulatory T-cell (CD25^+^ FoxP3^+^) induction, defined as RORγt^-^, RORγt^+^, or RORγt^+^ IL-17A^+^ regulatory T-cells (CD25^+^ FoxP3^+^), after 7 days in human PBMCs (n = 6 donors) stimulated with heat-inactivated *A. fumigatus* conidia (1 × 10^7^/mL) after 1 hour pre-incubation with 10 µg/mL αTLR2, or CoIgG. Data are represented as mean ± SEM. (**D**) IL-17A production after 7 days, IL-10 production after 24 hours and 7 days (n = 14 donors), regulatory T-cell (CD25^+^ FoxP3^+^) induction after 7 days, and IL-17A and RORγt expression within regulatory T-cells after 7 days in human PBMCs (n = 9 donors) stimulated with heat-inactivated *A. fumigatus* conidia (1 × 10^7^/mL) in the presence or absence of 10 µg/mL P3C or FSL1. (**E**,**F**) Different subsets within regulatory T-cell (CD25^+^ FoxP3^+^) induction, defined as RORγt^-^, RORγt^+^, or RORγt^+^ IL-17A^+^ regulatory T-cells (CD25^+^ FoxP3^+^), after 7 days in human PBMCs (n = 6 donors) stimulated with heat-inactivated *A. fumigatus* conidia (1 × 10^7^/mL) in the presence or absence of 10 µg/ml P3C. Data are represented as mean ± SEM. Abbreviations: *Af* = *Aspergillus fumigatus*; *CoIgG* = Control immunoglobulin (**G**); *αTLR2* = anti-TLR2 antibody; *P3C* = Pam3Cys-SKKKK; *FSL-1* = Pam2Cys-SKKKK. *p-value ≤ 0.05, **p-value ≤0.01, ***p-value ≤ 0.001.

As demonstrated previously^29^, blocking TLR2 before stimulating with *A. fumigatus* conidia resulted in a significant increase in IL-17A production (p = 0.0039 n = 9). However, no change in IL-10 production after 24 hours, and after 7 days was observed (Fig. 3A). Within the CD4^+^ population, the number of CD25^+^FoxP3^+^ T_reg_ cells significantly decreased with TLR2 blockade (p = 0.0117 n = 9), while a non-significant trend towards increased expression of T_H_17 cell-characteristics, i.e. RORγt^+^IL-17A^+^ within these cells was observed (p = 0.1875 n = 6) (Fig. 3B). Expression of T_H_17 cell-characteristics, i.e. RORγt^−^, RORγt^+^, and RORγt^+^IL-17A^+^, within CD25^+^FoxP3^+^ T_reg_ cells are depicted in Fig. 3C.

### TLR2 co-stimulation reduces expansion of regulatory T-cells with a T_H_17-like phenotype

To further dissect the role of TLR2 in *Aspergillus*-induced T_reg_ cells with pro-inflammatory T_H_17-like characteristics, strong TLR2 ligands, i.e. P3C (Pam3Cys-SKKKK; TLR2 and TLR1 heterodimer agonist) and FSL-1 (Pam2Cys-SKKKK; TLR2 and TLR6 heterodimer agonist), were combined with *A. fumigatus* stimulation assays. Co-stimulation of TLR2 with P3C and FSL-1 resulted in a significant decrease in IL-17A production after 7 days (p = 0.0002 n = 14) and a significant increase in IL-10 production after 24 hours (p = 0.0039 and p = 0.0254 n = 10), but not after 7 days (Fig. 3D). No significant change in the expansion of CD25^+^FoxP3^+^ T_reg_ cells was observed upon co-stimulation of TLR2 with P3C, while the population of CD25^+^ FoxP3^+^ T_reg_ cells expressing the T_H_17 RORγt^+^IL-17A^+^ phenotype was significantly smaller (p = 0.0313 n = 7) (Fig. 3E). Expression of T_H_17 cell-characteristics, i.e. RORγt^−^, RORγt^+^, and RORγt^+^IL-17A^+^, within CD25^+^FoxP3^+^ T_reg_ cells are depicted in Fig. 3F.

### CTLA4 regulates the induction of *Aspergillus*-induced regulatory T-cells with a T_H_17-like phenotype

**Figure 4:**
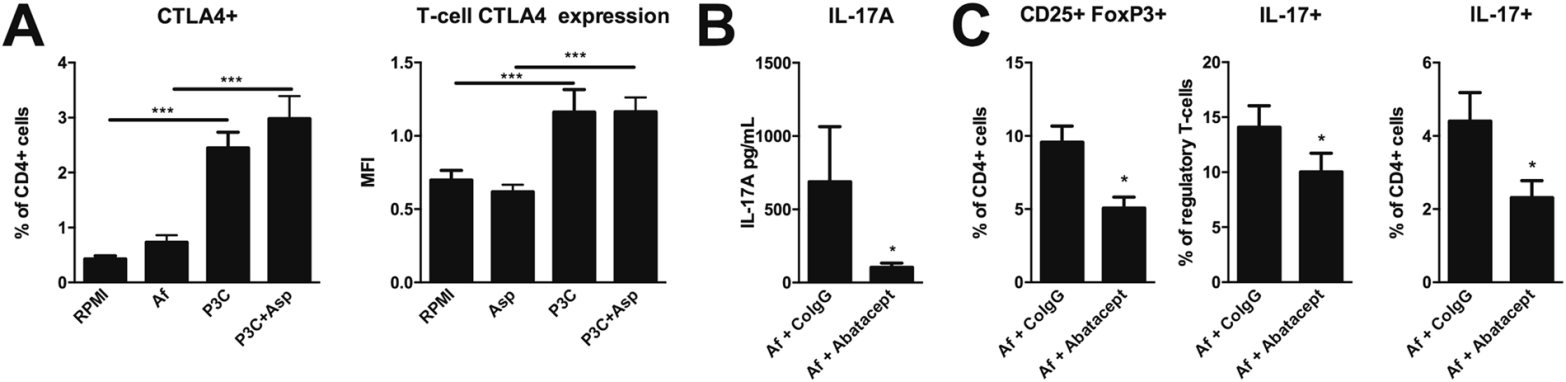
CTLA4 regulates *Aspergillus*-induced regulatory T-cells with aT_H_17-like phenotype. (**A**) Percentage of CTLA4 expressing CD4^+^ T-cells and Mean fluorescence intensity (MFI) of CTLA4 staining following stimulation of human PBMCs (n = 13 donors) with heat-inactivated *A. fumigatus* conidia (1 × 10^7^/mL), 10 µg/mL P3C or a combination of both. (**B**) IL-17A concentration in culture supernatants of human PBMCs (n = 6 donors) stimulated with heat-inactivated *A. fumigatus* conidia (1 × 10^7^/mL) after 1 hour pre-incubation with 24 µg/mL Abatacept, or CoIgG. (**C**) Regulatory T-cell (CD25^+^FoxP3^+^) induction after 7 days in human PBMCs (n = 7 donors) stimulated with heat-inactivated *A. fumigatus* conidia (1 × 10^7^/mL) after 1 hour pre-incubation with 24 µg/mL Abatacept, or CoIgG. T_H_17 cell (IL-17A + ) induction after 7 days in human PBMCs stimulated with heat- inactivated *A. fumigatus* conidia (1 × 10^7^/mL) after 1 hour pre-incubation with 24 µg/mL Abatacept, or CoIgG. IL-17A^+^ regulatory T-cell induction after 7 days in human PBMCs stimulated with heat- inactivated *A. fumigatus* conidia (1 × 10^7^/mL) after 1 hour pre-incubation with 24 µg/mL Abatacept, or CoIgG. Data are represented as mean ± SEM. Abbreviations: *P3C* = Pam3Cys-SKKKK; *Af* = A*spergillus fumigatus*; *CoIgG* = Control immunoglobulin G. *p-value ≤ 0.05, **p-value ≤ 0.01,***p-value ≤ 0.001.

One of the most potent molecules that regulates the induction of pro-inflammatory T-cell subsets is CTLA4. To determine whether there is a role for CTLA4 in controlling the induction of IL-17A-expressing regulatory T-cells by *Aspergillus*, we investigated these cells in a patient with CTLA4 deficiency. The CTLA4 deficient patient showed slightly reduced induction of CD25^+^FoxP3^+^ regulatory T-cells and a similar induction of IL-17A^+^ T_H_17 cells, compared to the healthy control. T_H_17 cell-characteristics, i.e. IL-17A^+^, within CD25^+^FoxP3^+^ T_reg_ cells, were substantially more induced by the patient compared to the healthy control (Supplementary Figure 2). To assess whether the capacity of TLR2 to reduce the expansion of regulatory T-cells with a T_H_17 phenotype was due to modulation of CTLA4, the capability to induce CTLA4 through TLR2 signalling was investigated. Activation of TLR2 by the agonist P3C resulted in a significant upregulation of CTLA4 expressing CD4^+^ T-cells (p = 0.0049 n = 12). Similarly, co-stimulation of TLR2 with *A. fumigatus* significantly upregulated the induction of CTLA4 expressing CD4^+^ T-cells, compared to stimulation with *A. fumigatus* (p = 0.0005 n = 12) (Fig. 4A). Soluble CTLA4–Ig (Abatacept) is known to inhibit the T-cell co-stimulatory molecule B7-1 (CD80), similar to endogenous CTLA4^30^. Using soluble CTLA4, we validated whether increased CTLA4 levels could reduce *Aspergillus*-induced CD25^+^FoxP3^+^ regulatory T-cells with a T_H_17 phenotype. Addition of CTLA4-Ig to human PBMCs resulted in a decreased IL-17A production after 7 days (p = 0.0234 n = 7) (Fig. 4B). Also, induction of the number of CD25^+^FoxP3^+^ regulatory T-cells expressing a T_H_17 phenotype, i.e. IL-17A^+^, CD25^+^FoxP3^+^ regulatory T-cells and IL-17A^+^ T_H_17 cells significantly decreased upon addition of CTLA4-Ig (p = 0.0343, p = 0.0343 and p = 0.0469 n = 7) (Fig. 4D).

## Discussion

In this study we investigated whether *A. fumigatus*-induced T_reg_ cells exert a pro-inflammatory phenotype, and if this pro-inflammatory phenotype can be reprogrammed to the “classical” anti-inflammatory T_reg_ phenotype. Human PBMCs exposed to *A. fumigatus* conidia showed induction of T_reg_ cells with pro-inflammatory T_H_17-like characteristics. Induction of classical (CD25^+^FoxP3^+^) T_reg_ cells and (IL-17A^+^RORγt^+^) T_H_17 cells was also significantly increased and related with increased production of IL-10 and IL-17A. These results indicate that *A. fumigatus* can indeed induce T_reg_ cells with a pro-inflammatory T_H_17-like phenotype, which potentially could contribute to detrimental IL-17-mediated immunopathology.

Although the IL-17A axis plays an important role in the protective immunity against fungal pathogens such as *A. fumigatus* ^31^, it has been demonstrated that in some cases IL-17A mediated immune responses overwhelm this protective effect, promoting infection and impairing antifungal immunity^13, 32^. Diseases such as CPA and ABPA are characterized by a persistent hyper inflammatory state with massive influx of neutrophils and eosinophils^12, 15^, which may be attributed to exaggerated T_H_17 responses. T_reg_ cells are potent suppressors of T_H_17 cells^17^, yet this suppressive effect is annulled when regulatory T-cells also acquire T_H_17 characteristics. In our study, we observed that the reduction of T_reg_ cells with a T_H_17 phenotype, either through TLR2 stimulation or Abatacept, correlated with a reduction of the T_H_17 cytokine IL-17. Since it is technically difficult to determine the origin of IL-17 in culture supernatants we cannot ascertain that this reduction of IL-17A is strictly due to a reduction of IL-17^+^ T_reg_ cells.

TLR2, together with TLR4 and TLR9, is an important Toll-like receptor in the host defence against *Aspergillus* (reviewed in refs 26, 33). The observation that splenocytes of *Tlr2* ^−/−^ mice have an increased *A. fumigatus*-induced production of IL-17A, directed us towards a possible suppressive role for TLR2 in the induction of IL-17 responses. In addition, many studies have demonstrated a critical role for TLR2 in the induction of regulatory T-cells^27, 28, 34^. We found that blocking TLR2, while stimulating with *A. fumigatus* conidia, exaggerated the induction of T_reg_ cells with pro-inflammatory T_H_17-like characteristics. Reversely, we observed that TLR2 co-stimulation not only increased the induction of regulatory T-cells, but also dampened the induction of regulatory T-cells with a T_H_17 phenotype. We suggest that altered TLR2 signalling may be involved in the aberrant induction of T_reg_ cells with pro-inflammatory T_H_17-like characteristics. Whether alterations in TLR2 signalling, pathogen- or host-related, are actually involved in IL-17 mediated immunopathology needs further investigation. An attractive approach would be to investigate whether common genetic variations that slightly alter TLR2 function are associated with detrimental IL-17-mediated inflammation in aspergillosis.

TLR2 activating therapy is currently under consideration as a potential immunotherapy to increase the number of regulatory T-cells, reducing T_H_2 mediated hypersensitivity^35^, and increasing anti-tumour capacity^36^. However, whether such therapies are attractive to employ for treatment of aspergillosis remains to be determined. Experimental evidence highlights that TLR2 plays an important role in the antifungal host response against *Aspergillus*. TLR2 deficient mice for example show an impaired recruitment of neutrophils to the site of infection^37^, and a higher lethality and fungal burden^33^. Interestingly, these mice also show an increased T_H_2 responses compared to WT mice upon stimulation with *A. fumigatus* ^37^. This T_H_2 response, associated with ABPA, suppresses the protective T_H_1 response^38^. In line with our data it is tempting to speculate that the TLR2 deficient mice are more susceptible to aspergillosis due to modulation of T_reg_ cells, as TLR2 mediated induction of T_reg_ cells is crucial for maintaining pro-inflammatory responses and promoting fungal clearance in aspergillosis^33, 37^. Several studies have clearly demonstrated a protective role of T_reg_ cells in fungal infection^39, 40^, and that TLR2 plays a crucial role in maintaining this population^28, 34^. It can however not be excluded that TLR2 has multiple lines of action in host defence against aspergillosis, such as induction of pro-inflammatory responses crucial for recruitment and activation of innate immune cells that are responsible for clearing the fungi from the lungs.

T_reg_ cells partly exert their anti-inflammatory function through contact dependent mechanisms such as CTLA4^20^. PBMCs of one patient who was deficient for CTLA4 were stimulated with *A. fumigatus*. Interestingly, a higher induction of T_reg_ cells with a pro-inflammatory T_H_17-like phenotype was observed, compared to the healthy control. In the field of rheumatology, therapeutics such as Abatacept (CTLA4–Ig) are often administered to reduce pro-inflammatory responses in which the T_H_17 – T_reg_ balance shifts towards the T_H_17 cell-response^41^. Therefore, such therapeutics might help reverse T_reg_ cells with pro-inflammatory T_H_17-like characteristics back to their “classical” anti-inflammatory phenotype. We examined the role of Abatacept in preventing the induction of T_reg_ cells with T_H_17-like characteristics, and a significantly decreased induction of T_reg_ cells with T_H_17-like characteristics was seen. This indicates that Abatacept has the potential to hamper the induction of T_reg_ cells with pro-inflammatory T_H_17-like characteristics. These results also indicate that CTLA4 might play a role in hampering the induction of T_reg_ cells with pro-inflammatory T_H_17-like characteristics, and warrants further investigation to explore whether Abatacept could be used as a targeted therapy in patients with aspergillosis that are suffering from infection-induced immunopathology.

We observed that TLR2 stimulation increases the number of CTLA4 expressing CD4 T-cells and the level of expression on these cells. Separately we demonstrated that either TLR2 stimulation or CTLA4 could reduce the induction of T_reg_ cells with pro-inflammatory T_H_17-like characteristics. The fact that TLR2 signalling boosts CTLA4 expression could suggest that TLR2 modulates the T_H_17 phenotype of *Aspergillus*-induced T_reg_ cells through CTLA4. It should be noted that *Aspergillus* itself did not significantly induce CTLA4 expression. It is therefore tempting to suggest that *Aspergillus* induces such a pro-inflammatory T_reg_ phenotype due to the fact that it fails to induce CTLA4 expression, but does induce the inflammatory mediators required for induction of pro-inflammatory T-cell responses.

Collectively, our study demonstrates that *A. fumigatus* is capable of inducing regulatory T-cells with a pro-inflammatory T_H_17-like phenotype. In addition, we found that TLR2 and CTLA4 play a role in regulating the induction of these cells. These findings could pave the way for novel therapeutic approaches that target T_reg_ cells with pro-inflammatory T_H_17-like characteristics in order to return them to their natural immune regulatory state.

## Methods

### Healthy volunteers and patients

Blood was collected from healthy volunteers and patients by venous blood puncture, after informed consent was obtained. One patient that was deficient for CTLA4 was included, and for those experiment the healthy control was age (±5 years) and sex-matched. All experiments were performed and conducted in accordance to Good Clinical practice, the Declaration of Helsinki, and the approval of the Arnhem-Nijmegen Ethical Committee (nr.2010/104).

### Aspergillus fumigatus

*A. fumigatus* V05-27, a previously characterized clinical isolate^42^, was used for all stimulations. Resting conidia were heat-inactivated, for 1 h at 65 °C and were checked for viability on Sabouraud agar. Conidia were stored at −80 °C until use. A concentration of 1 × 10^7^/mL was used in the experiments, unless otherwise indicated.

### Patter recognition receptor blockers and ligands

P3C (10 μg/mL) (Pam3Cys-SKKKK; TLR2/TLR1 heterodimer ligand, EMC microcollections, Tübingen, Germany), FSL-1 (1 μg/mL) (Pam2Cys-SKKKK; TLR2/TLR6 heterodimer ligand, EMC microcollections, Tübingen, Germany), and anti-TLR2 blocking antibody/control IgG (10 μg/mL) (eBioscience, Halle-Zoersel, Belgium) were used for pre-incubation and co-stimulation with heat-inactivated *A. fumigatus* conidia. Abatacept (CTLA4–Ig, 24 μg/mL) (inhibits the T-cell co-stimulatory molecule B7-1 (CD80), Orencia, Mulgrave, Australia) was used for pre-incubation with heat-inactivated *A. fumigatus* conidia.

### Isolation and stimulation of peripheral blood mononuclear cells (PBMCs)

Blood was diluted in phosphate buffered saline (PBS) (1:1) and fractions were separated by Ficoll (Ficoll-Paque Plus; GE healthcare, Zeist, The Netherlands) density gradient centrifugation. Cells were washed twice with PBS and resuspended in Roswell Park Memorial Institute medium (RPMI) 1640 Dutch modification culture medium (Life Technologies/ Invitrogen, Breda, The Netherlands) supplemented with 50 μg/mL gentamicin, 2 mM Glutamax, and 1 mM pyruvate (Life Technologies). Cells were counted using a particle counter (Beckmann Coulter, Woerden, The Netherlands) after which, the concentration was adjusted to 5 × 10^6^/mL. PBMCs were plated in 96-well round-bottom plates (Corning) at a concentration of 5 × 10^5^/mL in a total volume of 200 µL. The samples were stimulated with *A. fumigatus* heat inactivated conidia (1 × 10^7^/mL), with or without TLR2 blockers/ligands or Abatacept, or remained unstimulated for either 24 hours or 7 days at 37 °C with 5% CO_2_. After stimulation, supernatants were collected and stored at −20 °C until cytokine assays were performed. Cell pellets were used for flowcytometry.

### qPCR

RNA was isolated using TRIzol reagent (Invitrogen) according to the protocol supplied by the manufacturer, and RNA was converted into cDNA using iScript cDNA synthesis kit (Biorad, Hercules Ca). Quantitative real-time PCR (qPCR) was performed using power SYBR Green PCR master mix (Applied Biosystems, Carlsbad, CA) and the following primers: GAPDH FWD-5′-AGGGGAGATTCAGTGTGGTG-3′ REV-5′-CGACCACTTTGTCAAGCTCA-3′, RORγt FWD-5′-TGAGAAGGACAGGGAGCCAA-3′ REV-5′-CCACAGATTTTGCAAGGGATCA-3′ and FoxP3 FWD-5′-CTGCCCCTAGTCATGGTGG-3′ REV-5′-CTGGAGGAGTGCCTGTAAGTG-3′. PCR was performed using an Applied Biosystem StepOne PCR system using PCR conditions 2 min 50 °C, 10 min 95 °C, followed by 40 cycles at 95 °C for 15 sec, and 60 °C for 1 min. RNA expression was corrected for differences in loading concentration using the signal of housekeeping protein GAPDH.

### Cytokine measurements

IL-17A (R&D Systems, Mineapolis, M﻿N) and IL-10 (Sanquin, Amsterdam, the Netherlands) were measured using commercially available ELISAs according to the protocols that were supplied by the manufacturers. Mouse IL-17A was assessed in splenocyte stimulations using the Luminex multiplex platform (Millipore, Billerica, MA).

### Flowcytometry

For assessment of regulatory T-cells with T_H_17 characteristics, PBMCs were re-stimulated for 6 hours with phorbol 12-myristate 13-acetate (PMA) (50 ng/mL) (Sigma-Aldrich, Zwijndrecht, the Netherlands), Ionomycin (1 μg/mL) (Sigma-Aldrich), and GolgiPluginhibitor (1 µL/mL) (BD Biosciences, Breda, The Netherlands) after 7 day stimulation with *A. fumigatus*. Cells were stained extracellularly using Phycoerythrin(PE)–conjugated anti-CD4 (clone RPA-T4; ITK Diagnostics BV, Uithoorn, the Netherlands) or Phycoerythrin- Texas Red-X(ECD)-conjugated anti-CD4 (clone SFCI12T4D11; Beckman Coulter) and Phycoerythrin-Cyanine7–conjugated anti-CD25 (clone BC96; eBioscience) antibody. Subsequently, the cells were fixed and permeabilized with Cytofix/Cytoperm solution (eBioscience) according to the protocol that was supplied by the manufacturer. Following permeabilization, the cells were stained intracellular with Alexa Fluor 647–conjugated anti–IL-17A (clone N49-653; BD Biosciences), PE-conjugated RORγt (clone AFKJS-9; eBioscience, only used in non-patient experiments) and Alexa Fluor 488-conjugated FoxP3 (clone PCH101; eBioscience) according to the protocols supplied by the manufacturers.

For assessment of CTLA4 expression, PBMCs were stained extracellularly using Fluorescein Isothiocyanate (FITC)–conjugated anti-CD4 (clone RPA-T4; ITK Diagnostics BV) and PE- conjugated anti-CD152 (CTLA4) (clone 14D3; eBiosciences).

The cells were measured on a FC500 flow cytometer (Beckman Coulter) and the data were analysed using CXP analysis software v2.2 (Beckman Coulter).

### *Ex vivo* Stimulation of WT and Tlr2−/− murine splenocytes

Animal studies were carried out in accordance with guidelines and regulations approved by St. Jude Children’s Research Hospital Committee on Use and Care of Animals (protocol no 482-100265-1-/13). Wild-type (WT) and *Tlr2* knockout (*Tlr2* ^−/−^) C57Bl/6 mice were bred and maintained in the St. Jude Children’s Research Hospital, Memphis, TN, USA. Spleens were homogenized in 0,4 μM cell strainer (BD) and the cell number was adjusted to 1 × 10^7^/mL. The cell suspensions (500 μL/well) were placed in a 24 well plate (Corning) and incubated with culture medium or *A. fumigatus* conidia for 1 or 5 days at 37 °C and 5% CO_2_.

### Statistical analysis

Data of PBMC stimulation with either RPMI or *A. fumigatus* were analysed using the Wilcoxon signed rank test. P-values < 0.05 were considered statistically significant. Data are shown as scattered dot plots, columns with mean ± standard error of mean (SEM), or as a Venn diagram. All data were analysed using GraphPad Prism v5.0. The proportional Venn diagram was drawn using the eulerAPE application v2.0.3^43^.

## Electronic supplementary material

Supplementary Figures
