# Supplementary material for: Toll-like receptor 2 induced cytotoxic T-lymphocyte-associated protein 4 regulates Aspergillus-induced regulatory T-cells with pro-inflammatory characteristics
Source: Sci Rep. 2017 Sep 13;7:11500. doi: 10.1038/s41598-017-11738-4 (PMC5597613; doi:10.1038/s41598-017-11738-4)
Supplement: Supplementary file 1 — Supplementary Figures [file 41598_2017_11738_MOESM1_ESM.doc]

**Supplementary information**

**TLR2 induced CTLA4 regulates *Aspergillus*-induced regulatory T-cells with pro-inflammatory characteristics**

Ruud P.H. Raijmakers, Evelien G.G. Sprenkeler, Floor E. Aleva, Cor W.M. Jacobs, Thirumala-Devi Kanneganti, Leo A.B. Joosten, Frank L. van de Veerdonk, Mark S. Gresnigt^1*^


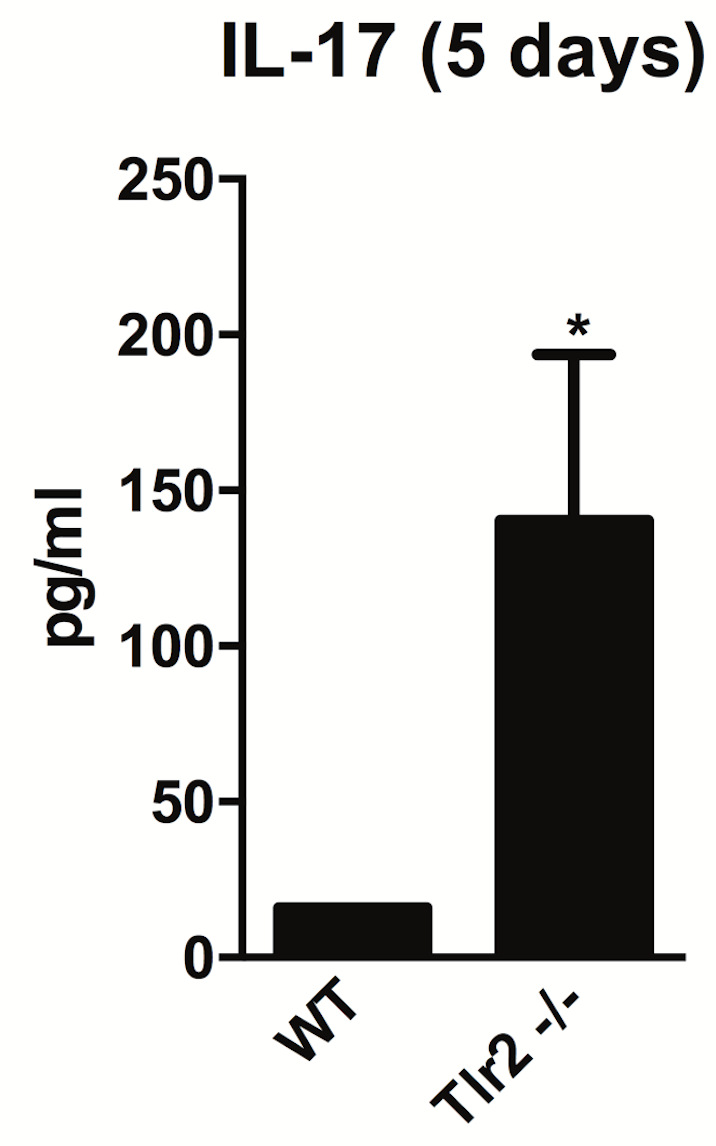


**Supplementary figure 1. Splenocytes of *Tlr2*^-/-^ mice produce higher levels of IL-17A than those of WT mice**

IL-17A concentration in culture supernatants of WT, and *Tlr2*^-/-^, murine splenocytes (n = 5 / group) stimulated for 5 days with heat-inactivated *A. fumigatus* conidia (1×10^7^/ml). Abbreviations: *WT* = wild type; *Tlr2 -/-* = Toll-like receptor 2 knockout. p-value ≤0.05

**
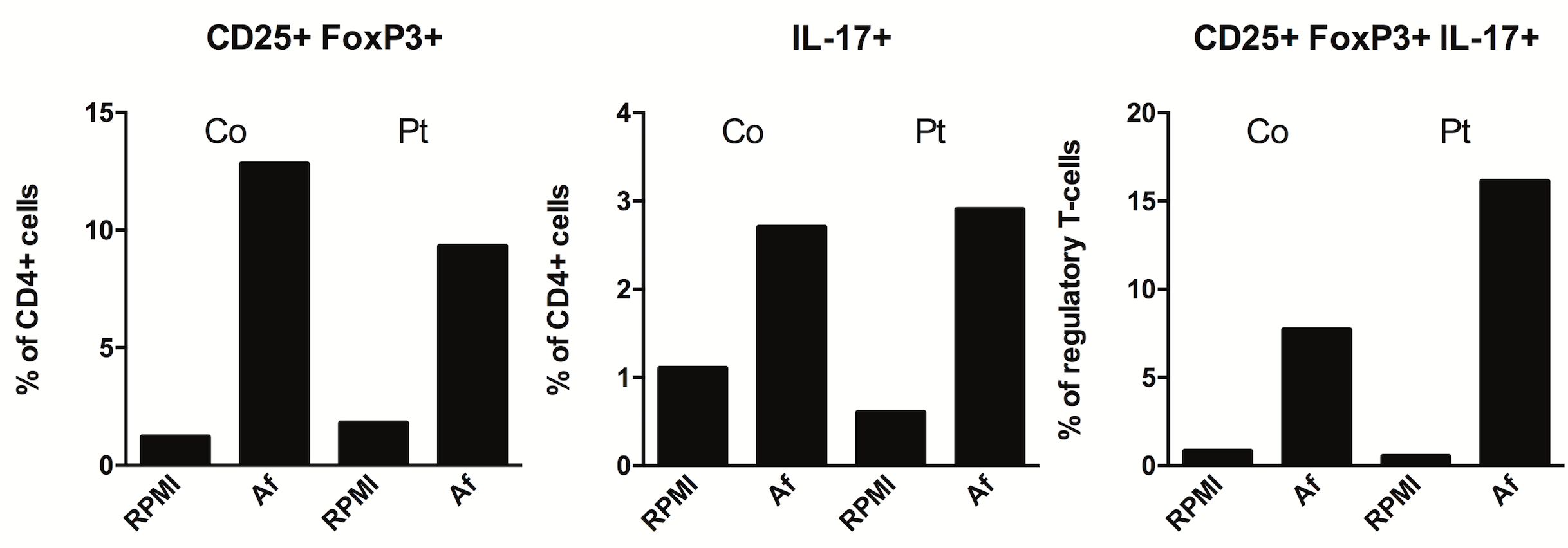
**

**Supplementary figure 2.**

Regulatory T-cell (CD25^+^ FoxP3^+^), T_H_17 cell (IL-17A^+^), and IL-17A^+^ regulatory T-cell induction after 7 days in PBMCs of a CTLA4 deficient patient and healthy control, stimulated with either RPMI or heat-inactivated *A. fumigatus* conidia (1×10^7^/ml).
